# Supplementary figures and images for: Optimal intrusion detection for imbalanced data using Bagging method with deep neural network optimized by flower pollination algorithm
Source: PeerJ Comput Sci. 2025 Mar 17;11:e2745. doi: 10.7717/peerj-cs.2745 (PMC11935772; doi:10.7717/peerj-cs.2745)

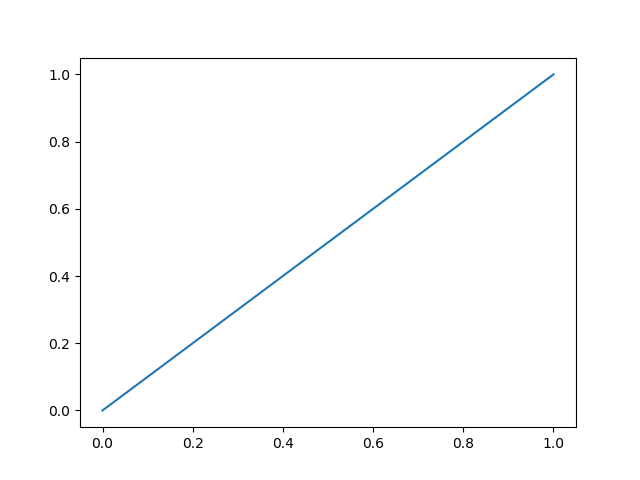

Supplement: Supplemental Information 2 [file peerj-cs-11-2745-s002.zip › cs-101223-IDS-optDNN-1/IDS-optDNN/easyplot.png]
